# Molecular Epidemiology of *Entamoeba*: First Description of *Entamoeba moshkovskii* in a Rural Area from Central Colombia

**DOI:** 10.1371/journal.pone.0140302

**Published:** 2015-10-14

**Authors:** Myriam Consuelo López, Cielo M. León, Jairo Fonseca, Patricia Reyes, Ligia Moncada, Mario J. Olivera, Juan David Ramírez

**Affiliations:** 1 Departamento de Salud Pública, Facultad de Medicina, Universidad Nacional de Colombia, Bogotá, Colombia; 2 Grupo de Investigaciones Microbiológicas–UR (GIMUR), Facultad de Ciencias Naturales y Matemáticas, Universidad del Rosario, Bogotá, Colombia; University of Texas Medical Branch, UNITED STATES

## Abstract

**Background:**

*Entamoeba histolytica*, *E*. *dispar* and *E*. *moshkovskii* are the most frequent species described in human infection where *E*. *histolytica* is the only true pathogen. The epidemiology of this infection is complex due to the absence of a routine exam that allows a correct discrimination of the *Entamoeba* species complex. Therefore, molecular methods appear as the unique epidemiological tool to accomplish the species discrimination. Herein, we conducted a cross-sectional study to determine the frequency of *Entamoeba* species infections in a group of asymptomatic individuals from a rural area in central Colombia.

**Methodology/Principal Findings:**

A total of 181 fecal samples from asymptomatic children under 16 years old from the hamlet La Vírgen, Cundinamarca (Colombia) that voluntarily accepted to participate in the study were collected. The fecal samples were examined by light microscopy and DNA-extracted, subsequently submitted to molecular discrimination of *E*. *dispar/E*. *histolytica/E*. *moshkovskii* infection based on a multiplex PCR assay targeting the 18S rRNA fragment. To confirm the species description, twenty samples were randomly submitted to DNA sequencing of the aforementioned fragment. By direct microscopic examination, frequency of the complex *E*. *histolytica/E*. *dispar/E*. *moshkovskii* was 18.8% (34/181). PCR showed a frequency of 49.1% (89/181), discriminated as 23.2% (42/181) that were positive for *E*. *dispar*, 25.4% (46/181) for *E*. *moshkovskii* and 0.55% (1/ 181) for *E*. *histolytica*. Also, mixed infections were detected between *E*. *dispar* and *E*. *moshkovskii* at 4.42% (8/181) of the samples. Molecular barcoding confirmed the diagnosis depicted by the multiplex PCR assay.

**Conclusions/Significance:**

This is the first description of *E*. *moshkovskii* in Colombia and the second report in South-America to our knowledge. Our results suggest the need to unravel the true epidemiology of *Entamoeba* infections around the world, including the real pathogenic role that *E*. *moshkovskii* may have.

## Introduction

Amoebiasis is the parasitism caused by *Entamoeba histolytica*, the only pathogenic species among the amoebas that inhabit in the human digestive tract and other organs [1]. It is one of the most prevalent parasitic diseases worldwide, particularly in developing countries where sanitation is insufficient [2]. The genus *Entamoeba* includes six species: *E*. *histolytica*, *E*. *dispar*, *E*. *moshkovskii*, *E*. *bangladeshi*, *E*. *poleki*, *E*. *coli* and *E*. *hartmanni*. The first three species are morphologically similar but with different biochemical and genetic features [3]. Previously, it was considered that about 500 million people were infected with *E*. *histolytica* worldwide. However, this frequency is overestimated due to the use of light microscopy instead of molecular tools able to differentiate the species [2, 4]. Asymptomatic presentation is the most common clinical form of amoebiasis (around 80–90% of the cases) and might be caused by three independent species: The pathogenic amoeba *E*. *histolytica*, *E*. *dispar* considered as a commensal and *E*. *moshkovskii* in which the pathogenic potential is still under discussion. This premise reflects the need to understand the molecular epidemiology of these species in endemic countries [5].

Recent studies have reported the presence of *E*. *moshkovskii* in fecal samples from humans in different countries such as United States, Italy, Iran, Turkey, Bangladesh, India, Australia and Brazil [3, 6, 7, 8, 9, 10]. The role that *E*. *moshkovskii* plays in human health is controversial, some authors still consider this species as a commensal but recent studies in India and Bangladesh have identified this species as the only likely pathogen in individuals with gastrointestinal clinical manifestations including dysentery [5, 7, 8, 11]. However, in these patients, no studies of viral or bacterial agents were conducted to rule out other pathogens or potential pathology agents.

At present, the prevalence of infection discriminated by species of *Entamoeba* is barely known [12, 13, 14, 15]. To address this problem, different molecular methodologies have been used to distinguish the three species (*E*. *histolytica*, *E*. *dispar* and *E*. *moshkovskii*) and have proved to be useful as an epidemiological and surveillance tool [3, 12, 15, 16, 17]. In studies deploying these methodologies, predominance of infection with *E*. *dispar* has been reported, while *E*. *histolytica* infection looks to be less frequent. Although the distribution of species seems to change according to the region studied [12, 5, 18]. In Latin American countries: Nicaragua, Brazil and Ecuador have reported a higher prevalence of *E*. *dispar* compared to *E*. *histolytica* [19, 20, 21]. However, countries such as Venezuela and Mexico have reported that *E*. *histolytica* is more frequent than *E*. *dispar* [22, 23, 24]. In Colombia there is no data (by molecular methods) on the prevalence of these three amoebas in humans and also if there exist the presence of *E*. *moshkovskii*. Therefore, the objective of this study was to differentially detect the presence of *E*. *histolytica*, *E*. *dispar* and *E*. *moshkovskii* by PCR in stool samples from school children in a rural community of Cundinamarca, Colombia.

## Materials and Methods

### Ethical statement

We obtained 181 fecal samples from asymptomatic children under 16 years old from the hamlet La Vírgen, Cundinamarca (Colombia) that voluntarily accepted to participate in the study. The children were physically examined to verify their asymptomatic status. La Virgen hamlet is located in the department of Cundinamarca at 4° 45´ north latitude and 74° 32´west longitude with an altitude of 1050 meters above sea level [25, 26]. The overall percentage of unsatisfied basic needs (UBS) is 58%. A parent or guardian of any child participant of this study provided written informed consent on their behalf. The ethical clearance of this study was followed by the ethics of Helsinki declaration and resolution No. 008430 of 1993 from the Ministry of Health from Colombia and “El Código del Menor”. The study protocol was approved by the ethics committee from the faculty of Medicine of the Universidad Nacional de Colombia under the Number 0045763.

### Fecal samples collection, microscopic diagnosis and DNA extraction

The fecal samples were collected in plastic recipients, labeled and conserved in refrigerated boxes. The samples were divided in two parts: one part was fixed in a proportion (1:4) in ethanol 70% and stored at -20°C for DNA extraction. The other part was used for conducting Kato-Katz, modified Richie-Frick method and direct microcopy examination for diagnosis of intestinal parasites [27, 28]. From each sample, 250 mg were submitted to DNA extraction using the QIAmp DNA Stool Mini Kit (Qiagen, Hilden, Germany) according to manufacturer´s instructions. Genomic DNA was preserved at -20°C until analysis.

### *E*. *histolytica/dispar/moshkovskii* PCR discrimination and statistical analyses

Differential identification of *E*. *histolytica/E*. *dispar/E*. *moshkovskii* was performed by a simple PCR protocol described by Hamzah et al., 2006 that targets the small rRNA subunit. [29]. Positive controls used for carrying out the PCR were reference strains listed as follows: *E*. *histolytica* HM1 strain, *E*. *dispar* ISS strain and *E*. *moshkoskii* SAW760 Laredo strain were kindly provided by Dr. Graham Clark, London School of Hygiene and Tropical Medicine. As negative control was used DNA extracted from a sample of stool from a healthy individual, whom tested negative by light microscopy, Gal / GalNAc lectin determination and PCR [30].

The four oligonucleotides used in PCR were described by Hamzah et al., 2006 [29]. The mixture of the three oligonucleotides allowed the specific amplification of genomic DNA of *E*. *histolytica*, *E*. *dispar* and *E*. *moshkovskii*. The sequence of the sense oligonucleotide (ENTAF) represents the central region of the gene encoding the small subunit ribosomal RNA, conserved in all three species of *Entamoeba*; antisense oligonucleotides EhR, EmR and EdR are specific for *E*. *histolytica*, *E*. *moshkovskii* and *E*. *dispar* respectively. The primers sequences: ENTAF: 5'-GAG CAC AGC ATG AGC GAA AT-3 '; EhR: 5'-GAT CTA GAA CTC ACA CTT ATG T-3 '; EdR: 5'-CAC CAC TCC CTA CTA TTA DC-3, 'EmR, 5'-TGA GCC CCA GAG GAG ACA T-3'. The combination of oligonucleotides generated specifically products of 166 bp for *E*. *histolytica* DNA, 752 bp for *E*. *dispar* DNA, and 580-bp for *E*. *moshkovskii* DNA [31]. A total of 20 samples were submitted to DNA sequencing of the partial region of the 18S rRNA gene for the confirmation of the diagnosis depicted by PCR. The PCR products were digested with EXOSAP (Affymetrix, USA) and sequenced by the dideoxy-terminal method in an automated capillary sequencer (AB3730, Applied Biosystems) by both strands in Macrogen (Korea). The sequences were submitted to BLASTn for similarity search with *Entamoeba* sequences deposited on the databases. The resulting sequences were edited in MEGA 5.0 and aligned using ClustalW 1.8 with reference sequences from *E*. *histolytica* (FJ888636), *E*. *dispar* (KJ719489), *E*. *moshkovskii* (KJ719489), *E*. *bangladeshi* (JQ412862), *E*. *polecki* (FR686399), *E*. *coli* (FR686448) and *E*. *hartmanni* (FR686382) retrieved from GeneBank [32, 33]. All edited sequences were deposited in GenBank and assigned accession numbers (Under submission). A maximum composite likelihood (MCL) analysis using a Tamura-3 parameter was run in RaxML Phylogeny.fr platform. To evaluate the robustness of the nodes in the resulting phylogenetic tree, 1000 bootstrap replicates were performed.

Bivariate and multivariate analyses by logistic regression model were conducted to determine the factors (age and sex) potentially related to the infections and parasitic co-infections by estimation of odds ratio (OR) and confidence intervals of 95%. In all cases, p value < 0.05 was considered significant. The analysis was done using the Stata version 10.0 (Stata Corporation, College Station, USA). The Kappa (κ) test assessed by the scale [34] was used to measure the correlation between microscopy methods for the complex *E*. *histolytica/ E*. *dispar/E*. *moshkovskii* and PCR for differential diagnosis of the three species. The relationship between the variables of age, gender, and other co-infections with the prevalence of the species *E*. *histolytica/E*. *dispar/E*. *moshkovskii* measured by PCR was determined by testing Χ^2^. Statistical analyses were performed using the EPIDAT v3.1 program (Directorate of Public Saúde Xeral, Xunta de Galicia (Spain) -PAHO).

## Results

### Prevalence of *E*. *histolytica*, *E*. *dispar*, *E*.*moshkovskii* according to different diagnostic methods

When compared by age and gender, no significant associations were observed in the infection by *E*. *dispar*, *E*. *moshkovskii* and/or *E*. *histolytica* (Table 1). However, infection with *E*. *moshkovskii* in the group of 12–15 years is observed as a factor risk compared to the age group of 5–8 years old. By direct microscopic examination, the frequency of the complex *E*. *histolytica/E*. *dispar/E*. *moshkovskii* was 18.8% (34/181). PCR showed a frequency of 49.2% (89/181), discriminated as 23.2% (42/181) positive for *E*. *dispar*, 25.4% (46/181) for *E*. *moshkovskii* and 0.55% (1/ 181) for *E*. *histolytica*. Also, mixed infections were detected between *E*. *dispar* and *E*. *moshkovskii* at 4.42% (8/181) of the samples. Finally, the correlation between PCR and light microscopy was moderate on the scale of Altman: Kappa 0.451 (p <0.05; 95% CI: 0342–0560).

**Table 1:** Bivariate and Multivariate analysis of the *E*. *dispar* and *E*. *moshkovskii* infections and other co-infections in relation to age and sex.

Parasite	Associations	Bivariate analysis			Multivariate analysis		
		Coefficient	OR (CI 95%)	*P*	Coefficient	OR (CI 95%)	*P*
*E*. *histolytica*	**Sex**						
	Male	Ref	Ref		Ref	Ref	
	Female	-0.49	0.95 (0.47–1.91)	0.891	-0.01	0.98 (0.48–2.01)	0.971
	**Age**						
	5–8	Ref	Ref		Ref	Ref	
	9–11	-1.41	0.87 (0.39–1.93)	0.73	-0.14	0.87 (0.37–1.97)	0.739
	12–15	0.14	1.14 (0.47–2.79)	0.765	0.13	1.15 (0.48–2.79)	0.764
*E*. *moshkovskii*	**Sex**						
	Male	Ref	Ref		Ref	Ref	
	Female	-0.55	0.58 (0.29–1.13)	0.113	-0.51	0.59 (0.29–1.20)	0.149
	**Age**						
	5–8	Ref	Ref		Ref	Ref	
	9–11	-0.05	0.94 (0.42–2.12)	0.89	0.05	1.05 (0.46–2.41)	1.05
	12–15	0.78	2.18 (0.93–5.10)	0.074	0.79	2.21 (0.95–5.23)	0.069
*E*. *dispar*	**Sex**						
	Male	Ref	Ref		Ref	Ref	
	Female	-0.08	0.92 (0.42–1.97)	0.82	-0.1	0.90 (0.41–1.96)	0.798
	**Age**						
	5–8	Ref	Ref		Ref	Ref	
	9–11	-0.11	0.89 (0.18–4.41)	0.891	-0.01	0.98 (0.19–4.98)	0.988
	12–15	1.27	3.57 (0.21–58.62)	0.372	1.14	3.15 (0.17–55.41)	0.432
Co-*infection`A*. *lumbricoides—T*. *trichiura*	**Sex**						
	Male	Ref	Ref		Ref	Ref	
	Female	0.79	2.22 (0.56–8.67)	0.25	1.01	2.76 (0.68–11.2)	0.151
	**Age**						
	5–8	Ref	Ref		Ref	Ref	
	9–11	-0.77	0.46 (0.08–2.61)	0.383	-0.96	0.38 (0.07–2.19)	0.28
	12–15	0.77	2.15 (0.54–8.53)	0.275	0.76	2.14 (0.53–8.62)	0.282
Co-*infection Hookworms—T*. *trichiura*	**Sex**						
	Male	Ref	Ref		Ref	Ref	
	Female	1.07	2.92 (0.59–14.42)	0.189	1.25	3.52 (0.69–17.93)	0.13
	**Age**						
	5–8	Ref	Ref		Ref	Ref	
	9–11	-0.46	0.62 (0.10–3.87)	0.615	-0.69	0.49 (0.07–3.15)	0.46
	12–15	0.81	2.27 (0.48–10.70)	0.3	0.89	2.26 (0.47–10.85)	0.308

Ref = reference; OR = odds ratio; CI = Confidence Interval

### DNA sequence analysis

**Fig 1:**
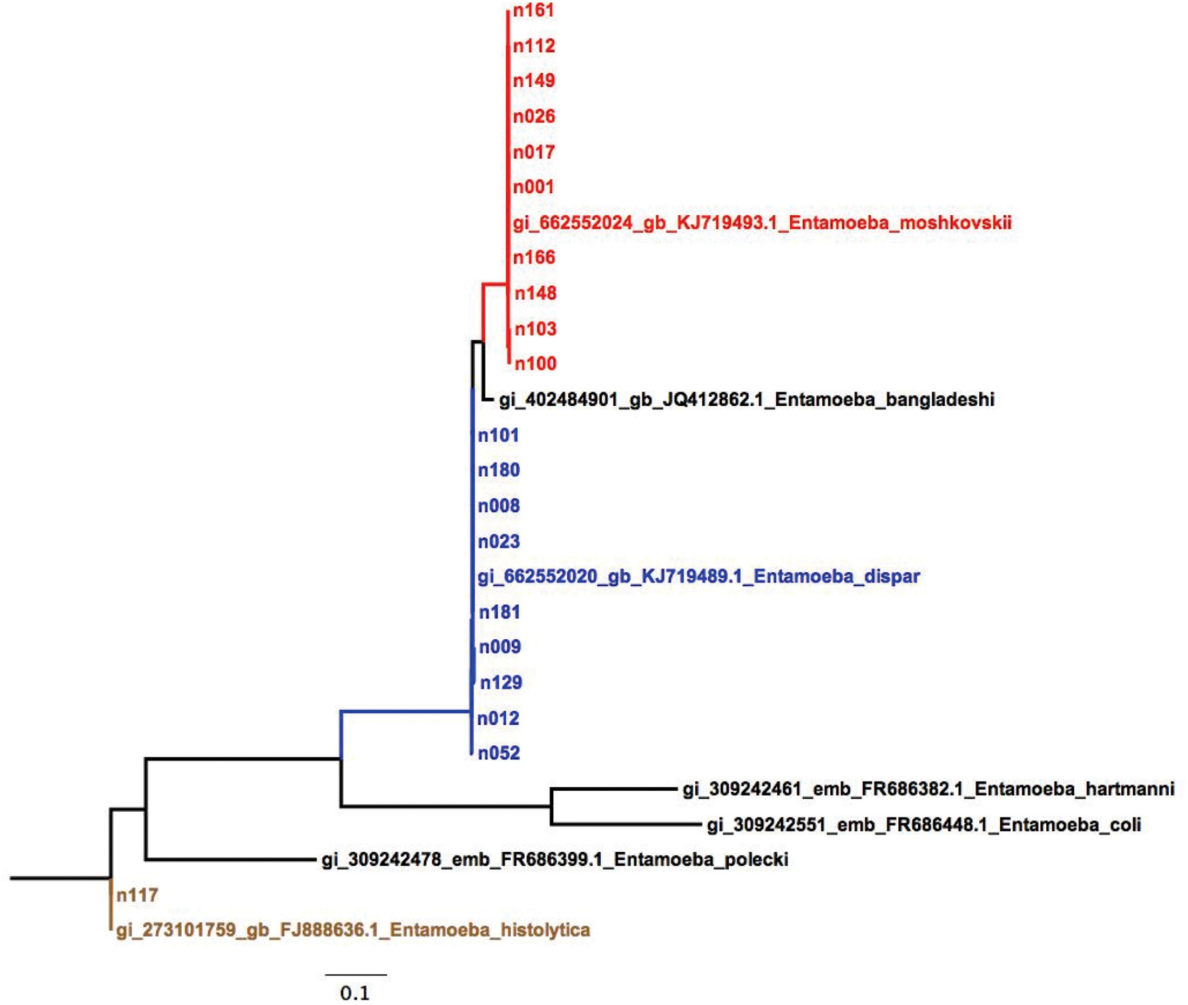
Maximum Composite Likelihood reconstruction of phylogenetically related 18S rRNA sequences from Amoeba species including 20 DNA sequences from the samples analyzed.

18S rRNA sequences were obtained from 20 samples (one *E*. *histolytica*, nine *E*. *dispar* and ten *E*. *moshkovskii*). Sequences were deposited on GenBank under the accession numbers KT825974-KT825993. The BLAST results showed a 100% similarity for one *E*. *histolytica* sample, seven *E*. *dispar* and seven *E*. *moshkovskii* samples. Two samples from *E*. *dispar* (n103 and n100) and three from *E*. *moshkovskii* (n009, n129 and n181) showed a similarity of 97%. A ML tree was built according to the 18S sequences to confirm the detection of *E*. *dispar*, *E*. *histolytica* and *E*. *moshkovskii* (Fig 1). The results showed the correct assignment of the *Entamoeba* species and the congruence with the PCR assay. Albeit, the first and novel description of *E*. *moshkovskii* in Colombia.

### Presence of co-infections

Among the patients included in the study, 85.1% (154/181) were diagnosed with at least one intestinal parasite. Twenty-five percent (45/181) of individuals were infected by one parasite, 26.5% (48/181) with 2 parasites and 30.9% (56/181) with 3 or more parasites. In total, the frequency of intestinal parasites in collected stool samples were: *Ascaris lumbricoides* (7%), *Trichuris trichiura* (14%), Hookworm (15%), *Hymenolepis nana* (1%), *Chilomastix mesnili* (3%), *E*. *coli* (34%), *Endolimax nana* (45%), *Iodamoeba butschlii* (7%), *Blastocystis* (34%), *E*. *hartmanni* (3%) and *Giardia duodenalis* (13%). Associations of co-infections with *E*. *dispar* and *E*. *moshkovskii* are shown in Table 2. No statistical association between the co-infections with the frequency of the *Entamoeba* species was observed (p = 0.123).

**Table 2:** Presence of co-infections (protozoan of helminth parasites).

	Patients infected by *E*. *moshkovskii* (n = 46)	Patients not infected by *E*. *moshkovskii* (n = 135)	Patients infected by *E*. *dispar* (n = 42)	Patients not infected by *E*. *dispar* (n = 139)
**Protozoans**				
*Giardia duodenalis*	5	9	4	10
*Blastocystis spp*	15	31	19	23
*Entamoeba coli*	1	3	3	1
*Entamoeba hartmanni*	1	0	4	0
*Endolimax nana*	19	44	18	45
*Iodamoeba butschilli*	3	10	6	7
**Helminths**				
*Ascaris lumbricoides*	2	4	4	2
*Trichiuris Trichiura*	0	4	3	1
Hookworms	6	19	5	19

## Discussion

Amoebiasis is a diagnostic challenge, clearly due to the existence of three morphologically indistinguishable species leading to the overdiagnosis of the parasitism produced by *E*. *histolytica*. In this study, by light microscopy the most commonly method employed for diagnosis, 34 samples were found positive for the complex *E*. *histolytica/E*.*dispar/E*.*moshkovskii* but only one patient presented infection by *E*. *histolytica*, 42 with *E*. *dispar* and 46 with *E*. *moshkovskii* when PCR was used as a diagnostic method. This test increased the sensitivity and specificity leading to an appropriate treatment for the infected individual. If the diagnosis is conducted only by microscopy medication had to be administered unnecessarily from the clinical standpoint. This reinforces the need for differential diagnosis of *E*. *histolytica* with the other species that form the complex because so far this is the only species of the genus *Entamoeba* that requires one level treatment action. On the other hand, resistance to metronidazole in the clinical context has been reported in cases of amebic liver abscess [35, 36]. This has been more difficult to demonstrate in cases of intestinal amoebiasis [37].

The finding of *E*. *dispar* as responsible for most of the infections of the complex *E*. *histolytica/E*. *dispar/E*. *moshkovskii* has occurred in different studies [21, 38] and in those studies that use molecular methods to split the complex has been the most prevalent species [18, 19, 20, 29, 39, 40]. The reported frequency of *E*. *dispar* by PCR in our study was 23.2% (42/181). This is consistent with specific national studies reporting the frequency of *E*. *dispar* ranging from 15%–17% [30, 41]. The finding of only one case of *E*. *histolytica* could be due to sample size as the reported prevalence in our country does not exceed 1.5% when antigen detection methods are used [30, 41]. Intriguingly, in our study the frequency of *E*. *moshkovskii* was a bit higher than *E*. *dispar* frequency suggesting that the circulation of this species is extant and not detected in previous examinations.

To our knowledge this is the second study in South America that sought to identify *E*. *moshkovskii* by PCR and is the first report of human infection by *E*. *moshkovskii* in Colombia [21]. Despite the mentioned above, the fact of detecting in our country by molecular methods *E*. *moshkovskii* in wastewater used for agriculture (unpublished data, pending response pairs) and the recent evidence that supports pathogenicity of this amoeba [42] suggests the likely possibility that this amoeba species is circulating in humans in our country. Also, we need further studies with larger samples to determine the epidemiology of this infection. This finding presents epidemiological significance as it is the first report of *E*. *moshkovskii* in Colombia and the second in South America and suggests the existence of this species as a possible emerging pathogen in developing countries. We corroborated these findings conducting DNA sequencing of the 18S rRNA markers observing 97–100% similarity with the *E*.*dispar*, *E*. *histolytica*, *E*. *moshkovskii* sequences from GenBank (Fig 1). Additionally, we detected a low amount of drift in our samples (n103, n100, n009, n129 and n181) that need to be further analyzed by high-resolution molecular markers.

Another factor to consider is that the species of *Entamoeba* that are not part of the complex *E*. *histolytica/E*.*dispar/E*.*moshkvoskii* as *E*. *hartmanii*, *E*. *bangladeshi* and *E*. *poleckii* sometimes can be morphologically similar to the afore-mentioned complex [3] and even for people trained in the diagnosis of amoebiasis can generate false positives. This problem is further accentuated in laboratories that are not reference centers where structures such as macrophages or lymphocytes may result in false positives [19]. The main advantage of using PCR is the ability to distinguish the different species of the complex, especially where there is a high prevalence of *E*. *dispar* or *E*. *moshkovskii*, making it the method of choice to understand the epidemiology of this infection [12]. However, in endemic areas this test cannot be applied routinely by the difficulties in extracting DNA from stool samples and the cost in time and money that led into the performance of this technique.

Microscopy still has an important place in the diagnosis context because identifies a variety of intestinal parasites that cause disease and often appear in co-infections with *E*. *histolytica*, *E*.*dispar*, *E*. *moshkovskii*, as was the case in our study where we detected *E*. *dispar* and *E*. *moshkovskii* infections with other protozoan pathogens (i.e. *Giardia*, *Blastocystis*) and helminths (i.e. *Ascaris lumbricoides*, *Trichiuris trichiura*, hookworms) but with no significant association (Table 1; Table 2). Regarding the risk factors for infection with *E*. *dispar* and *E*. *moshkovskii* neither age nor gender had relationship with this infection, which shows that the main determinants for acquiring this infection are sanitation of the population [43] (Table 1; Table 2). Within the limitations of this study is the selection by volunteer participants but when compared on the basis of the records of the schools of the population, showed no significant differences among students when compared by age, gender and place of origin. One would think that in a random selection, the number of participants with gastrointestinal symptoms, and therefore the number of cases of *E*. *histolytica* be increased so that only one patient with this parasite was found in this study. While it is important to note that when different authors have selected only patients with gastrointestinal symptoms, *E*. *dispar* is more prevalent than *E*. *histolytica* [44, 45, 46] and in many cases *E*. *histolytica* infection is presented in co-infection with other intestinal parasites or bacteria such as *Escherichia coli* or *Shigella* spp that may explain gastrointestinal symptomatology [45, 46].

### Conclusions

In conclusion, this study demonstrated that PCR is a useful diagnostic tool to determine the molecular epidemiology of *Entamoeba* species. Also, herein we report the first evidence of *E*. *moshkovskii* infection in humans from Colombia suggesting the need to pursue more studies to understand the transmission dynamics of this species and more importantly determine its pathogenic role.
